# Recent Advances in Metal-TADF Emitters and Their Application in Organic Light-Emitting Diodes

**DOI:** 10.3389/fchem.2020.00653

**Published:** 2020-07-31

**Authors:** Wai-Pong To, Gang Cheng, Glenna So Ming Tong, Dongling Zhou, Chi-Ming Che

**Affiliations:** ^1^State Key Laboratory of Synthetic Chemistry, HKU-CAS Joint Laboratory on New Materials, Department of Chemistry, The University of Hong Kong, Hong Kong, China; ^2^HKU Shenzhen Institute of Research and Innovation, Shenzhen, China

**Keywords:** TADF, gold, tungsten, palladium, silver, organic light-emitting diode

## Abstract

In this contribution, recent advances in new classes of efficient metal-TADF complexes, especially those of Au(I), Au(III), and W(VI), and their application in OLEDs are reviewed. The high performance (EQE = 25%) and long device operational lifetime (LT_95_ = 5,280 h) achieved in an OLED with tetradentate Au(III) TADF emitter reflect the competitiveness of this class of emitters for use in OLEDs with practical interest. The high EQE of 15.6% achieved in solution-processed OLED with W(VI) TADF emitter represents an alternative direction toward low-cost light-emitting materials. Finally, the design strategy of metal-TADF emitters and their next-stage development are discussed.

## Introduction

Thermally activated delayed fluorescence (TADF) materials have become a promising class of photo-functional materials with potential practical applications most exemplified in the field of organic light-emitting diodes (OLEDs). The majority of TADF materials reported to date are organic compounds having donor and acceptor moieties that give rise to emissive charge transfer (CT) excited states upon light excitation. By carefully positioning the donor(s) and acceptor(s), a small singlet-triplet energy gap (Δ*E*(S_1_-T_1_)) can be achieved, thereby allowing efficient reverse intersystem crossing (RISC) to singlet excited state and TADF to occur at room temperature. While numerous classes of molecular organic TADF materials have been reported, the diversity of metal complexes exhibiting TADF property is very limited. In this article, we discuss the recent advances on metal complexes which display TADF and their application in OLEDs.

## Recent Advances in Metal-TADF Complexes and Their Application in OLEDs

### Metal-TADF Light-Emitting Complexes

The burgeoning development of emitters for OLEDs started from the fluorescent metal complex 8-hydroxyquinoline aluminum (Alq_3_) (Tang and VanSlyke, 1987). Despite its high luminance, high electron mobility and stability, the efficiency of Alq_3_-based OLED is limited by its fluorescence nature. Theoretically, the upper limit of internal quantum efficiency (IQE) of a fluorescent OLED is about 25% as only singlet spin states can emit light in fluorescent emitters. In this regard, phosphorescent metal complexes have been attracting much attention considering the 100% potential IQE in electro-phosphorescence (Baldo et al., 1998; Ma et al., 1998). Typical phosphorescent emitters are heavy metal complexes such as those of Ru(II), Ir(III), Os(II), and Pt(II) with emission lifetimes (τ) ranging 1–100 μs due to significant mixing between the metal and ligand frontier molecular orbitals and the efficient spin-orbit coupling induced by the heavy metal atom. Because of their high luminescence efficiency and high stability, phosphorescent Ir(III) complexes have been widely used as emitting dopants in OLED industry (Baldo et al., 1999). Nonetheless, the earth abundance of iridium is the lowest among the metal elements, leading to sustainability concern. An alternative approach to harvest triplet excitons at room temperature is via TADF (Yersin et al., 2011; Uoyama et al., 2012). The key process in TADF is up-conversion from the lowest triplet excited state (T_1_) to the lowest singlet excited state (S_1_), which is then followed by fluorescence from S_1_ to the singlet ground state (S_0_). Therefore, a delicate trade-off between Δ*E*(S_1_-T_1_) and oscillator strength of S_0_-S_1_ transition is crucial to achieve TADF. Since the reports by Endo et al. (2009); Deaton et al. (2010), and also by Uoyama et al. (2012), a plethora of organic TADF molecules have been reported (Wong and Zysman-Colman, 2017; Yang et al., 2017) but examples of metal-TADF emitters are mostly limited to copper complexes (Czerwieniec et al., 2016; Leitl et al., 2016). Since Cu(I) complexes generally lack thermal and electrochemical stability, efforts have been directed to develop 2nd and 3rd row transition-metal-TADF emitters such as those of Pd(II), Ag(I), Au(I), and Au(III) to meet the stringent requirements of emitters for practical OLEDs (Li G. et al., 2019). Gratifyingly, high external quantum efficiencies (EQEs) of up to 27.5 and 25.0% have been achieved in OLEDs with Au(I) and Au(III) TADF emitters, respectively (Di et al., 2017; Zhou et al., 2020), reflecting Au-TADF emitters as an emerging new class of competitive, emissive dopant in OLED industry.

The first report on electroluminescence (EL) of Cu(I) complex appeared soon after the reports on OLEDs based on Pt(II) and Os(II) emitters (Ma et al., 1999a,b). A Cu(I) complex could exhibit phosphorescence or TADF depending on its Δ*E*(S_1_-T_1_) (Leitl et al., 2014). TADF could take place when the Δ*E*(S_1_-T_1_) is comparable to the thermal energy *k*_B_*T* that enables a dynamic equilibrium between S_1_ and T_1_. Several high-performance OLEDs based on Cu(I) complexes have been reported (Zhang et al., 2012, 2016; Cheng et al., 2015; Osawa et al., 2015; Volz et al., 2015; So et al., 2017; Hamze et al., 2019; Shi et al., 2019). For instance, Bräse, So, Baumann and co-workers reported EQEs of up to 23% for yellow-emitting OLEDs with an NHetPHOS-Cu(I) complex (NHetPHOS refers to a ligand having N-heterocycle and phosphine) as the emitter (Volz et al., 2015). Thompson reported a class of two-coordinate Cu(I) carbene amide complexes exhibiting photoluminescent quantum yields (PLQYs) up to 1.0 with τ of 1–2 μs. EQEs of OLEDs fabricated with these emitters reached 19.4% (Shi et al., 2019). Since the development of TADF Cu(I) complexes has been well-reviewed (Czerwieniec et al., 2016; Yersin et al., 2017; Liu et al., 2018; Li G. et al., 2019), we will focus in this contribution on recent advances of efficient TADF metal complexes other than Cu(I) complexes that have been applied as emitting dopants in OLEDs.

### Silver TADF Complexes and Their Application in OLEDs

**Figure 1:**
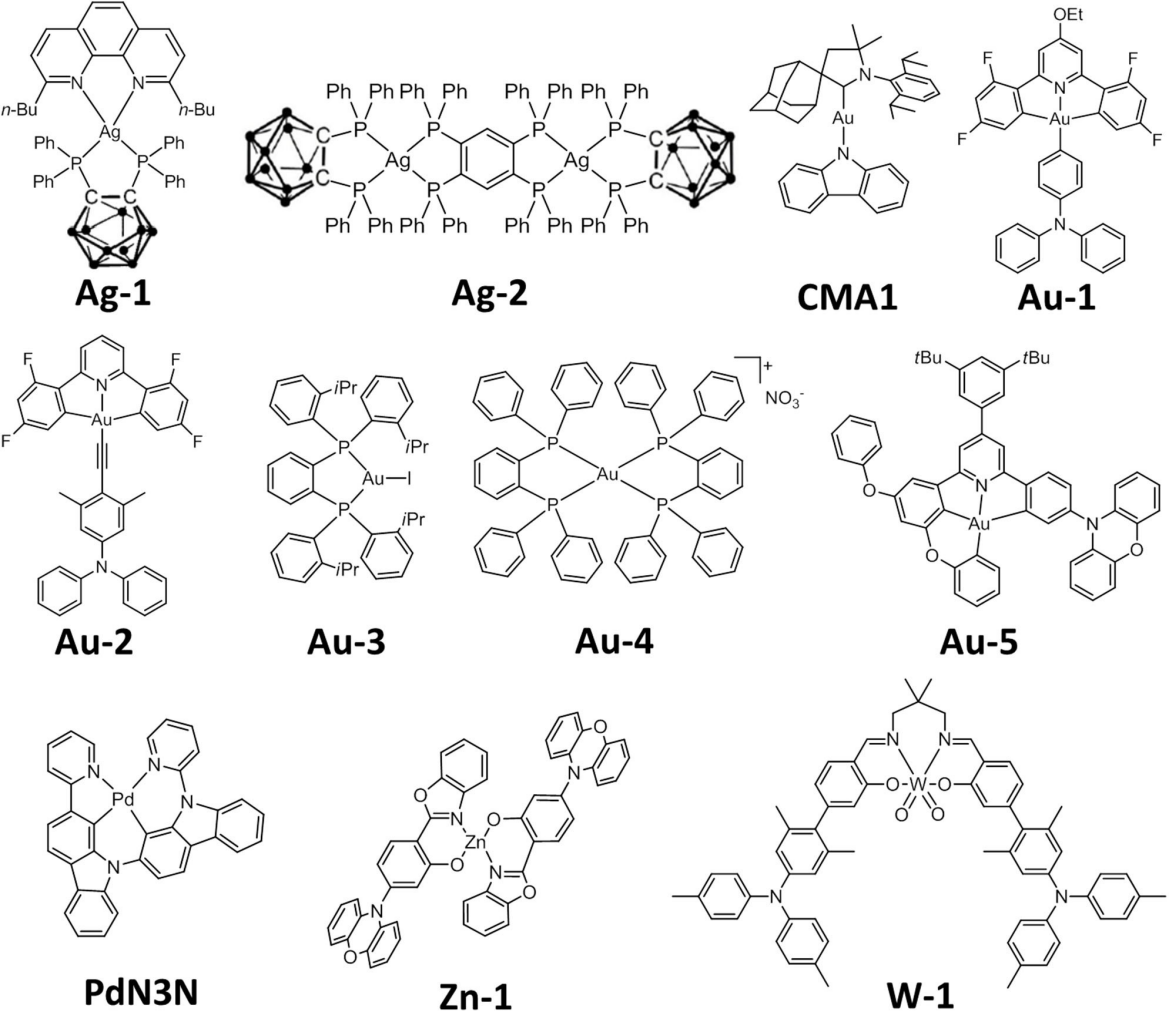
Chemical structures of selected metal-TADF complexes.

Several examples of Ag(I) TADF emitters have been reported recently. Lu discovered bis-bidentate tetraphosphine bridged binuclear Ag(I) halide complexes exhibiting TADF with PLQYs up to 0.98 and lifetime of 2.5–3.0 μs (Chen et al., 2016). Yersin reported a series of Ag(I) complexes supported by 1,10-phenanthroline and bis(diphenylphosphine)-nido-carborane (e.g., **Ag-1**, Figure 1) with PLQYs up to 1.0 and τ ranging 1.4–2.8 μs (Shafikov et al., 2017a,b). Replacing 1,10-phenanthroline with a bridging tetraphosphine ligand afforded a binuclear Ag(I) TADF complex **Ag-2** with PLQY of 0.70 and τ of 1.9 μs (Shafikov et al., 2018). A Ag(I) carbene amide complex also exhibits TADF with PLQY of 0.74 and τ of 460 ns in degassed toluene. OLEDs fabricated with this emitter showed EQEs up to 13.7% (Romanov et al., 2018).

### Gold TADF Complexes and Their Application in OLEDs

Gold is an attractive candidate for developing OLED emitters attributable to the high thermal stability endowed by strong gold-ligand bonds and its relatively high abundance among other noble metals in Earth's crust. Due to the electrophilicity/relatively high reduction potential of Au(III), Au(III) complexes often display ligand-centered emission having minute metal contribution. This results in small radiative decay rate constants (*k*_r_) of 10^2^-10^3^ s^−1^ and hence long τ (usually >10 μs), longer than those of typical Ir(III) and Pt(II) complexes by one or two orders of magnitude (Zhou et al., 2019). As long emission lifetimes would cause severe efficiency roll-off in OLEDs (Cheng et al., 2014), only a few of them could achieve decent EQE and low efficiency roll-off at the same time. Notably, the study on Au(I)-OLEDs was even rarer than those of Au(III)-OLEDs (Ma et al., 1999b,c). In this regard, the recent development of TADF gold complexes has made a remarkable turnaround. Linnolahti, Bochmann, Credgington and co-workers reported a series of 2-coordinate carbene–Au(I)–amides (CMAs) that consist of a cyclic (alkyl)(amino)carbene (CAAC) connected via Au(I) to a carbazolate or diphenylamide (Di et al., 2017). Computations revealed that Δ*E*(S_1_-T_1_) of **CMA-1** remains small (<800 cm^−1^) along the torsional coordinate, even at the coplanar conformation which has fluorescence rate of the order 10^7^ s^−1^, thus leading to a fast equilibration of the S_1_ and T_1_ excited states and a large *k*_r_ of 2.4 × 10^6^ s^−1^, PLQY of 0.83 and short τ of ~350 ns in neat film at 300 K (Föller and Marian, 2017; Conaghan et al., 2018). A maximum EQE of 26.3% was achieved in solution-processed OLEDs with **CMA1** as the emitter and high EQE of 24.5% was maintained at a luminance of 1,000 cd m^−2^. Nonetheless, there has been no report on the operational lifetimes of the aforementioned Au(I)/Cu(I)/Ag(I)-TADF OLEDs. In 2017, Che and co-workers identified TADF as the emission origin in several pincer Au(III) aryl emitters (To et al., 2017). The presence of diarylamino group on the monodentate aryl ligand, and its twisted geometry with respect to the cyclometalating ligand, results in TADF in **Au**-**1** (Figure 1). Based on variable temperature–emission lifetime measurements and DFT calculations, a Δ*E*(S_1_-T_1_) of 318 cm^−1^ was estimated for this complex. With short τ of 0.72 μs and high PLQY of 0.84 in room temperature, solution-processed OLEDs with **Au**-**1** showed EQE and luminance of up to 23.8% and 57,340 cd m^−2^, respectively. It is noted that both devices based on the Au(I) complex **CMA1** and the Au(III) complex **Au**-**1** were fabricated by solution-processed technique. In this regard, studies on EL of these gold complexes in vacuum-deposited devices were undertaken to determine if they have potential application in practical OLEDs. High maximum EQEs of 26.9 and 23.4% were achieved in vacuum-deposited OLEDs with Au(I) complex **CMA1** (Conaghan et al., 2018) and pincer Au(III) alkynyl complex **Au**-**2** (Figure 1; Zhou et al., 2019), respectively. Despite the slightly lower efficiency, the device lifetime (LT_95_) of the latter has been measured to be ~500 h at an initial luminance of 100 cd m^−2^, which is at least comparable to that of pincer Au(III) complex bearing deprotonated carbazole as auxiliary ligand reported by Li L.-K. et al. (2019). The improved thermal stability of Au(III) alkynyl complexes compared to the aryl ones is attributed to the stronger Au(III)-C_sp_ bond in the alkynyl counterparts. Au(I) complexes with diphosphine ligand(s) have also been reported to display TADF (Osawa et al., 2018). The crystalline solid of Au(I) diphosphine iodide (**Au-3**) displayed yellow photoluminescence with PLQY of 0.92 and lifetime of 9.0 μs. Its emission shows a red-shift of 20 nm, a decrease in PLQY to 0.74 and an increase of lifetime to 77 μs, corresponding to a reduction in *k*_r_ from 1.0 × 10^5^ s^−1^ to 9.6 × 10^3^ s^−1^ upon cooling from 293 to 77 K. Au(I) bis-diphosphine complex **Au-4** exhibits a high PLQY of 0.95 but with much shorter lifetime of 3.8 μs. The Δ*E*(S_1_-T_1_) of **Au-3** and **Au-4** were estimated to be 870 and 620 cm^−1^, respectively.

### Tetradentate Metal-TADF Emitters and Their Application in OLEDs

The aforementioned TADF Au(I) and Au(III) complexes were prepared by using two ligands. Since the stability of metal complexes could be increased by employing chelating ligands of higher denticity, the employment of tetradentate ligand with C-donor atom(s) is envisaged to improve the thermal stability and to restrict excited-state structural distortion of the resultant complex as exemplified in tetradentate Pt(II) and Pd(II) complexes (Vezzu et al., 2010; Cheng et al., 2013; Chow et al., 2016). Furthermore, it was suggested that an increase in the structural rigidity of emitters could suppress structural deformation upon S_1_-T_1_ transformation, which reduces Δ*E*(S_1_-T_1_) and leads to efficient TADF (Saigo et al., 2019). Therefore, the development of TADF Au(III) complexes supported by trianionic tetradentate ligands would be an appealing direction toward practical Au-OLEDs. A class of Au(III) complexes supported by N-bridged tetradentate ligand prepared by post-modification was reported by Wong et al. (2017). These complexes exhibit photoluminescence from triplet intraligand charge-transfer (ILCT) excited states with PLQY of up to 0.78 in thin films. Solution-processed OLEDs fabricated with these emitters showed EQE of up to 11.1%. Che and co-workers developed new strategies for synthesizing tetradentate Au(III) complexes with O-bridged/spiro-arranged C^∧^C^∧^N^∧^C ligand by microwave induced C-H activation (Zhou et al., 2020). By rationally varying the substituent(s) on the ligand, the emissive excited states of the Au(III) emitters are changed from triplet intraligand (^3^IL) excited states with *k*_r_ of ~10^3^ s^−1^, to TADF from ILCT excited states. These Au(III) TADF emitters show high thermal stability and PLQYs of up to 0.94 and τ down to 0.62 μs in degassed toluene. A vacuum-deposited OLED with **Au**-**5** as the emissive dopant showed maximum EQE of 25% and the EQE value maintained at 22% at a luminance of 1,000 cd m^−2^. Significantly, as listed in Table 1, at an initial luminance of 100 cd m^−2^, this device showed a much longer lifetime LT_95_ of 5,280 h. This value is at least 10-fold longer than those recorded with pincer Au(III) emitters (Li L.-K. et al., 2019; Zhou et al., 2019). This result highlights the advantage of using tetradentate ligand in the preparation of robust Au(III) TADF emitters for practical use. It also showcases tetradentate Au(III) TADF complexes as competitive candidate in OLED industry.

**Table 1:** Key performances of selected metal-TADF complexes.

**Complex**	**Fabrication method**	**EQE (%)**		**L^**d**^ (cd m^**−2**^)**	**CIE^**e**^ (x, y)**	**Lifetime^**f**^ (h)**
		**Max**.	**At 1,000 cd m^**−2**^**			
CMA1	SP^a^	26.3	25.2	44,700	0.26, 0.49	n. a.
	VD^b^	26.9	24.9	35,400	0.24, 0.42	n. a.
Au-1	SP^a^	23.8	16.5	33,740	0.27, 0.51	n. a.
Au-2	VD^b^	23.4	22.1	70,300	0.40, 0.55	500
Au-5	VD^b^	25.0	22.0	22,700	0.43, 0.54	5,280
PdN3N	VD^b^	20.9	7.0^c^	n. a.	0.30, 0.61	6,000^c^
Zn-1	VD^b^	19.6	n. a.	n. a.	n. a.	n. a.
W-1	SP^a^	15.6	9.7	16,890	0.49, 0.49	n. a.

a Solution process;

b Vacuum deposition;

c Estimated from the original reference;

d Maximum luminance;

e CIE coordinates;

f *LT_95_ at L_0_ = 100 cd m^−2^*.

Tetradentate ligand has also been used in preparing stable, luminescent Pd(II) complexes. Li and co-workers reported a tetradentate Pd(II) complex, **PdN3N**, which contains a C^∧^N cyclometalating moiety composed of 2-pyridyl-carbazole, where the T_1_ is localized, and also a donor-acceptor moiety of carbazole-carbazoyl-pyridine (Figure 1, Zhu et al., 2015). **PdN3N** exhibited both phosphorescence and TADF at room temperature with PLQY of 0.72. A maximum EQE of 20.9% and operational lifetime LT_90_ of 170 h at an initial luminance of 1,697 cd m^−2^ were achieved in the OLED with **PdN3N**. Nonetheless, the EQE of this device dropped to ~7.0% at a luminance of 1,000 cd m^−2^. Such severe efficiency roll-off is a result of the long τ of >100 μs for **PdN3N**, which limits the application of this kind of Pd(II) complexes in practical OLEDs. In addition, the color purity of the Pd-OLED is not good enough because of the wide EL spectrum resulting from dual emission from both phosphorescence and TADF.

### Inexpensive Metal-TADF Complexes and Their Application in OLEDs

The low earth abundance of noble metals (e.g., iridium, platinum) has stimulated a great interest to invent new classes of luminescent materials based on inexpensive, earth-abundant metals (Bizzarri et al., 2018; Wenger, 2018). Besides Cu(I) complexes, more examples of inexpensive metal-TADF emitters have been reported. Adachi and co-workers synthesized TADF materials based on zinc, magnesium and lithium having ILCT transition (Sakai et al., 2015). **Zn-1** (Figure 1) was the most efficient emitter among these complexes. The EQE of vacuum-deposited OLEDs based on **Zn-1** was up to nearly 20%. By employing terphenyl having carboxyl and diphenylamine groups as linkers, Adachi, Kabe and co-workers constructed a zirconium-based metal-organic framework exhibiting green TADF with PLQY of 0.30 under vacuum (Mieno et al., 2018).

Tungsten is another appealing candidate for developing luminescent metal-based materials because of its large spin-orbit coupling constant (2,433 cm^−1^) which facilitates intersystem crossing and significantly higher earth abundance than noble metals. Nonetheless, examples of air-stable tungsten complexes displaying strong photoluminescence are rare with the recent report on W(VI) *cis*-dioxo Schiff base and quinolinolate complexes by Yeung et al. (2017). One of these complexes exhibit PLQYs of up to 0.22 in thin film and was used as emitter to realize the first tungsten-OLED, though the maximum EQE achieved was only 4.79%. While the proof of principle has been demonstrated, the low PLQY and inferior performance data suggested that significant improvement in the photo-luminescent properties of luminescent W(VI) complexes is needed if the latter are to be used for practical applications. With a view to designing W(VI) complexes with competitive luminescent properties for OLED application, it is conceived that installation of diarylamino group(s) on the Schiff base ligand may change the emission origin to TADF (To et al., 2017; Zhou et al., 2019), thereby boosting *k*_r_ and improving PLQY. Che and co-workers described the first example of tungsten TADF emitter in 2019 (Chan et al., 2019). This study has demonstrated that the incorporation of diarylamino donor groups into the ligand scaffold changes the emissive excited state from long-lived ^3^IL ones to TADF arising from ILCT excited state, which effectively boosts PLQY of W(VI) Schiff base complex to 0.84 in thin film. The estimated *k*_r_ of **W-1** (Figure 1) is 4.2 × 10^5^ s^−1^, which is at least 100-fold larger than that of analogous W(VI) complexes without diarylamino group. DFT calculations revealed that the subtle change on the twisted angle between the diarylamino substituent and the phenolic moiety is the key that influences the excited state dynamics and modulates the singlet–triplet energy separation, leading to efficient TADF in complex **W-1**. Solution-processed OLEDs fabricated with **W-1** showed EQEs of up to 15.6%, which is a significant improvement compared to the previous work (EQE_max_ = 4.79%).

## Discussion

The variety and design strategy of metal-TADF emitters remain considerably scarce despite the recent discoveries on Au(I), Au(III), Ag(I), and Pd(II) TADF emitters. Summarizing from the recent findings, metal-TADF emitters are generally realized in complexes that are composed of electrophilic metal ion, such as those of Au(III), W(VI), and Pd(II). In these complexes, the emission origin is changed from phosphorescence (^3^LC) to TADF by adding donor groups to generate LLCT/ILCT excited states. The originally small *k*_r_ of these complexes allows an easy identification of whether TADF is operative because a 100-fold (or even more) increase in *k*_r_ would be observed when TADF takes place, as exemplified in Au(III) and W(VI) TADF emitters. This finding is similar to that of organic compounds which are known to show long-lived phosphorescence (*k*_r_ < 100 s^−1^). By incorporating donor-acceptor pair, TADF can be observed and the *k*_r_ of organic compounds can be increased significantly. Accordingly, it may be a challenge to realize TADF in Pt(II) and Ir(III) complexes due to their large phosphorescent *k*_r_ which facilitates efficient radiative decay via triplet excited state. Another consideration is that as the *k*_r_ of phosphorescent Pt(II) and Ir(III) are already large (10^5^-10^6^ s^−1^), switching the emission origin to TADF may not lead to a drastic increase in *k*_r_ and this renders the identification of whether TADF is occurring in these complexes highly challenging.

Compared to pure organic TADF emitters, τ of triplet excited states of metal-TADF complexes is much shorter, which could be advantageous for the operational stability of OLEDs based on metal-TADF emitters. Furthermore, with the use of rigid tetradentate ligands, a drastic improvement in terms of efficiency and device stability could be realized as that observed for tetradentate Au(III) TADF emitters. This finding calls for a more stringent ligand design for practical metal-TADF emitters in addition to pursuing high PLQY and short τ.

Besides, the questionable practicability of Cu(I) complexes in OLEDs triggers the investigation on TADF emitters based on other inexpensive metals. Although TADF Zn(II) and W(VI) complexes have shown high PLQY and EL efficiency, the improvement of operational lifetime of devices based on these complexes remains a formidable challenge. The deployment of robust and rigid ligands that would induce the occurrence of TADF could be the key to increase the practical potential of emitters based on earth-abundant metals.

## Author Contributions

All authors contributed to the writing of the manuscript.

## Conflict of Interest

The authors declare that the research was conducted in the absence of any commercial or financial relationships that could be construed as a potential conflict of interest.
